# Evaluation of treatment response in patients with recurrent grade 4 glioma using hyperpolarized [1-^13^C]pyruvate MRI

**DOI:** 10.21203/rs.3.rs-9238794/v1

**Published:** 2026-04-07

**Authors:** Sana Vaziri, Janine M Lupo, Adam W Autry, Marisa LaFontaine, Javier Villanueva-Meyer, Daniel B Vigneron, Nancy A Oberheim-Bush, Susan M Chang, Jennifer L Clarke, Yan Li

**Affiliations:** University of California San Francisco

## Abstract

Hyperpolarized (HP) [1-^13^C]pyruvate MRI can noninvasively detect dynamic metabolic activity in the brain. This study utilizes HP-[1-^13^C]pyruvate MRI to monitor treatment-related metabolism and evaluate early therapeutic response in twenty patients with recurrent grade 4 glioma undergoing cytotoxic, antiangiogenic, and targeted chemotherapies. Metabolic changes within the T2-lesion and normal-appearing white matter were compared among patients with similar treatments and/or outcomes. The lactate/pyruvate ratio increased by 14.3% at 1 month in the group whose therapy included bevacizumab, while it decreased by 13.1% at 2 months in the normal-appearing white matter of patients on everolimus. In the T2-lesion, patients on bevacizumab showed a 24.3% increase, whereas patients on everolimus showed a 7.6% decrease in normalized lactate/pyruvate ratios. Patients with shorter 6-month progression-free survival showed an average 9.6% increase in the lactate/pyruvate ratio as early as 1 month after treatment. This study demonstrates the potential of real-time metabolic imaging for response monitoring in patients with glioma.

## Introduction

Gliomas are the most common and malignant primary brain tumors in adults, with glioblastoma (GBM) being the most aggressive and prevalent form^1^. The latest WHO 2021 classification for grade 4 glioma now includes both isocitrate dehydrogenase (IDH)-mutant, grade 4 astrocytoma, and IDH-wildtype GBM categories^2^. For these grade 4 gliomas, standard-of-care treatment includes maximal surgical resection of the tumor followed by fractional radiation therapy (RT) and temozolomide (TMZ), a DNA alkylating agent^3^. Because patients eventually experience recurrence, a variety of second-line therapies are often integrated into the clinical management of recurrent disease as single or combined chemotherapeutic regimens. These therapies often include: (i) Lomustine (also known as CCNU, chloroethyl-cyclohexyl-nitrosourea), which is a standard second-line treatment after TMZ^4^; (ii) Everolimus, an mTORC1 inhibitor that decreases downstream protein synthesis and cell growth by blocking the mTOR pathway; or (iii) bevacizumab, an anti-angiogenic monoclonal antibody that inhibits the blood vessel formation. Despite these treatments, survival rates remain low, with fewer than 6% of patients living beyond five years after diagnosis, and a median overall survival of approximately 14–16 months^5-7^. In response to the limited efficacy of existing treatments, new therapeutic strategies are actively being developed to combat high-grade disease.

Standard clinical practice for planning treatment and response assessment for patients with glioma relies entirely on anatomical MRI-visible contrast-enhancing lesions (CEL) and/or T2 hyperintensity lesions (T2L)^8^, despite the fact that these features solely indicate structural changes caused by the breakdown of the blood-brain barrier or presence of fluid, respectively. Interpreting images, especially after therapy, can be challenging due to tumor heterogeneity and treatment-induced changes, highlighting the need for advanced MR techniques to inform therapy and clinical decisions. The known metabolic reprogramming—specifically, Warburg metabolic upregulation of glycolysis over oxidative phosphorylation^9^—suggests that incorporating metabolic imaging that can target this pathway could be useful for evaluating these patients.

Hyperpolarized carbon-13 (HP-^13^C) metabolic MR imaging provides a unique way to noninvasively and dynamically monitor metabolic pathways on a rapid timescale after the administration of the non-toxic, nonradioactive probe^10^. HP [1-^13^C]pyruvate is the most widely used ^13^C probe in human brain research, capable of detecting the Warburg effect in tumor^11^. Previous studies in high-grade gliomas demonstrated that HP [1-^13^C]pyruvate MRI can image, *in vivo*, the increased conversion of [1-^13^C]pyruvate to [1-^13^C]lactate compared to ^13^C-bicarbonate within tumors^12, 13^, and the ratio of those [1-^13^C]pyruvate to [1-^13^C]lactate was higher in recurrent tumors compared to treatment effects^13^. Interestingly, an increase in *k*_PL_, the conversion rate from pyruvate to lactate, was also detected in normal-appearing white matter (NAWM) using HP [1-^13^C]pyruvate MRI^14^ shortly after receiving antiangiogenic therapy, demonstrating its potential to identify early treatment-induced changes before they become visible on standard MRI.

The goal of this study was to characterize dynamic metabolic changes observed in both recurrent grade 4 lesions and NAWM in relation to treatment response during and after therapy. This involved monitoring patients with recurrent grade 4 glioma —either participating in a clinical trial (ID: NCT03681028, started on 12/19/2018) or seen in clinic— before and during chemotherapy for recurrent disease, using serial HP [1-^13^C]pyruvate MRI. We hypothesized that HP [1-^13^C]pyruvate MRI would detect early metabolic changes associated with response that preceded changes on standard MRI in both the lesion and, in cases where patients received antiangiogenic therapy, NAWM.

## Methods

### Patient Population

A total of 20 patients with recurrent grade 4 gliomas were enrolled in this IRB-approved study and provided informed consent. Tumor recurrence was confirmed through repeat surgery (19/20) or by the RANO criteria^8^ (1/20). Patients were then treated with different types of chemotherapy and monitored with serial MR exams that included both clinical ^1^H MRI and HP [1-^13^C]pyruvate MRI (N = 41). Of these patients, 12 had a baseline scan prior to receiving treatment for recurrence. When feasible, follow-up imaging was performed at approximately bimonthly intervals. The number and timing of follow-up scans varied across patients due to clinical scheduling constraints and external factors, including disruptions related to the COVID-19 pandemic. Of the 20 participants, 11 experienced disease progression (confirmed by subsequent surgery (N = 10) or RANO 2.0 criteria (N = 1)^8^) within 6 months of their baseline scan. Clinical characteristics and treatment regimens for each patient are summarized in Table 1.

Patients were divided into treatment-based groups. The first group, *BEV+*, consisted of patients who received bevacizumab as part of their treatment. The second group, *EVER+*, included those treated with everolimus in combination with other agents. The remaining patients who received multi-agent therapy with combinations of alkylating agents such as temozolomide (TMZ) or CCNU, along with other agents, like olaparib, abemaciclib, and afatinib, were grouped together into the *ALKYL+* group. Additionally, two patients served as controls: P19 was treated exclusively with TMZ, and P20 was treated only with CCNU. For the purpose of lesion analysis, patients were categorized according to their survival outcomes as either early progressors with a progression-free survival (PFS) less than 6 months, or late progressors with a PFS that exceeded 6 months.

### MR Data Acquisition

All MR studies were performed using a GE 3T MR750 scanner with a dual-tuned 24-channel ^13^C/8-channel ^1^H head array (Rapid Biomedical, Germany). The standard-of-care ^1^H MR clinical protocol included anatomical 3D pre and post-gadolinium (Gd) T1 IRSPGR (TR/TE/TI = 8/3/400 ms, 1.5 mm slices, 15° flip angle, 25.6cm x 25.6cm in-plane FOV, 256 × 256 matrix) and T2 weighted FLAIR (TR/TE/TI = 6250/138/1702 ms, 1.5mm slices, 25.6cm x 25.6cm in-plane FOV, 256 × 256 matrix) images.

The HP [1-^13^C]pyruvate samples were prepared using the clinical SPINlab system (General Electric, Niskayuna, NY). After passing the quality control, the solution was injected intravenously in patients (0.43 mL/kg at 5 mL/s)^14^, and then dynamic HP-^13^C data were acquired using a frequency-selective echo-planar imaging (EPI) sequence that independently excited each metabolite (TR/TE = 62.5ms/21.7ms, 8 slice, 20 dynamic timepoints, temporal resolution = 3-s, spatial resolution 1.5-cm isotropic with a 16 x 16 matrix size, flip angles constant *α*_pyruvate_/*α*_lactate_/*α*_bicarbonate_ = 20/30/30)^15^, except for one dataset, which was obtained with 2.0 cm isotropic spatial resolution. For 3 patients (P16, P19, and P20), HP-^13^C data were obtained from the same volume of interest using a template registered from the standard MNI152 space to the subject's space on the scan day via T1 images^16^. This approach enabled a direct voxel-wise comparison of HP-^13^C parameters across different time points. The other data acquisitions were prescribed axially.

#### Dynamic HP [1-^13^C]pyruvate MRI Processing

Raw dynamic EPI data were pre-whitened, channel-combined, and denoised as described previously^17-20^. The resulting dynamic metabolite maps for [1-^13^C]pyruvate, [1-^13^C]lactate, and [1-^13^C]bicarbonate were temporally summed to generate area-under-the-dynamic curve (AUC) metabolite maps. Noise standard deviations for dynamic metabolite images were estimated from the background using the brain mask generated from applying FSL’s Brain Extraction Tool^21^ on the T1 post-Gd image. Voxel-wise apparent signal-to-noise ratio (aSNR) maps were calculated for each metabolite using the denoised AUC images. SNR masks were created to filter out voxels with aSNR less than 5. After SNR masking of individual metabolites, ratio maps for lactate-to-pyruvate (Lac/Pyr), bicarbonate-to-pyruvate (Bic/Pyr), and bicarbonate-to-lactate (Bic/Lac) were derived from AUC maps. To facilitate comparison of metabolite signals across subjects and time points, voxel-wise raw AUC maps were transformed into percentile rank maps restricted to brain voxels. For each percentile map (Pyr%, Lac%, and Bic%), AUC values from brain-masked voxels were ranked using a tied ranking procedure that accounted for identical values, then normalized by the total number of brain voxels (without SNR filtering), and scaled to a 0–100 percentile range^13^. The resulting percentile rank maps will be referred to as Pyr%, Lac%, and Bic%. Voxel-wise apparent rate constants for pyruvate-to-lactate conversion (*k*_PL_) and pyruvate-to-bicarbonate conversion *k*_PB_ were quantified from dynamic metabolite data using an inputless kinetic model^14, 22^. Modeling error masks for *k*_PL_ and *k*_PB_ maps were created by filtering voxels with modeling error greater than 30% and 50% of the estimated value, respectively.

### Standard H MRI Image Processing & Region Definition

Automated gray and white matter segmentation was performed on the T1 pre-Gd images^23, 24^. White matter masks were refined by first aligning the T1 pre-Gd images using a non-linear registration^25^ to the AAL3 atlas^26^ and then removing the cortex. The T2L and CEL were semi-automatically segmented from the 3D FLAIR and T1 post-Gd images, respectively, using 3D Slicer^27^. The non-enhancing lesion (NEL) was then defined as the T2L minus the CEL. Similarly, NAWM was defined by subtracting any of the T2L that overlapped with the automatically-segmented white matter mask. Regions of interest (ROIs) defined at ^1^H image resolution were then mapped to the lower-resolution ^13^C matrix using a mutually exclusive labeling scheme: ^13^C voxels containing at least 30% of the ^1^H T2L were classified as ^13^C T2L. Of the remaining ^13^C voxels outside the T2L, those with at least 50% overlap with ^1^H NAWM were classified as ^13^C NAWM voxels. Subsequent lesion analyses at ^13^C resolution focused solely on T2L voxels, as the CEL volumes were relatively small for reliable serial comparison at the ^13^C spatial resolution in this study population.

### Data Analysis

Lesion volumes (CEL and NEL) were measured at the native ^1^H resolution for each patient scan. Percentile rank parameters (%Pyr, %Lac, %Bic) were summarized^13^ within each ROI (NAWM and T2L, calculated at ^13^C resolution voxels) by computing the median value across all voxels within the region for each patient scan. After applying SNR masks and modeling error masks, metabolite ratios and conversion rates were summarized in a similar manner. For lesion analyses, T2L values were then normalized by the corresponding patient-specific median NAWM value for each parameter. Group-level statistics were then calculated by taking the mean and standard deviation of these patient-wise median (and NAWM-normalized T2L) values across subjects within each group at each time point.

## Results

### Changes in Lesion Volume

**Figure 1:**
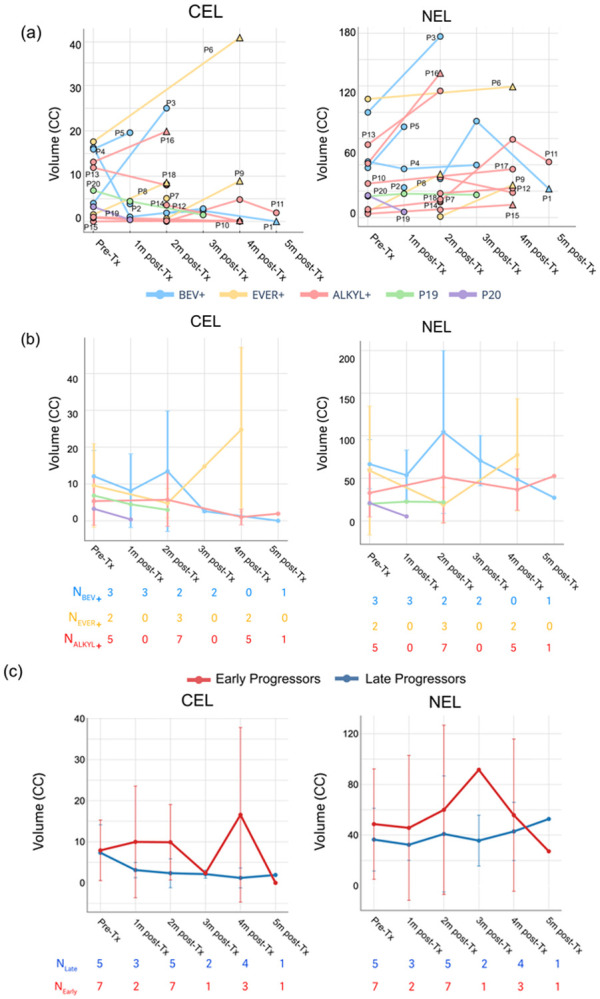
Lesion volumes over the course of the study. (a). Longitudinal trajectories (relative to the start of treatment) for individual patient ROI volumes for CEL (*left*) and NEL (*right*) in cubic centimeters(cc). Each line represents a single patient, with markers indicating measured volumes at scan time points. Circle markers indicate clinically stable time points, while triangles denote confirmed progression time points. (b-c) Group-averaged percent change in CEL (left) and NEL (*right*) volumes relative to pre-treatment baseline. Values represent the mean percent change across patients within each treatment group (b) or in the PFS group (c) at each follow-up time point. Error bars denote the standard deviation when more than one patient contributed data at a given time point; error bars are omitted when only a single patient was available (*N*).

Changes in lesion volume per patient after treatment are shown in Fig. 1a. Since most patients (19/20) had undergone surgical resection before treatment, the CEL volume was relatively small at baseline (median = 5.4 cm^3^, [0.0-17.6], N = 1 had no CEL), while the NEL volumes varied among patients (median = 39.7 cm^3^, [3.53–112.6]). Group averages by treatment category are shown in Fig. 1b. Compared to the pre-treatment baseline, changes in CEL and NEL volumes varied across treatment groups. Group averages by survival status (early vs late progressors) are presented in Fig. 1c. Despite differences among treatment groups, patients with longer PFS (PFS > 6 months) generally exhibit slower early post-treatment growth, especially in the CEL.

### Early Treatment Effects of Metabolism in the NAWM

**Figure 2:**
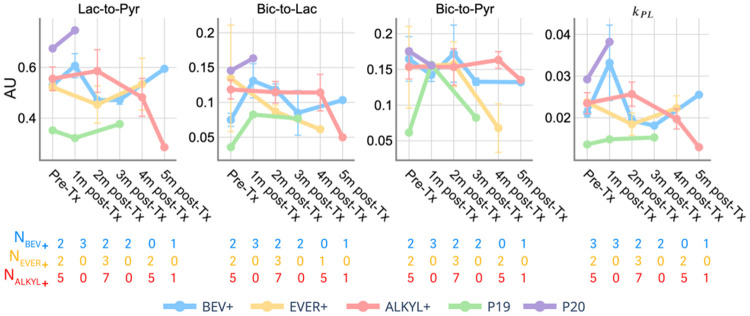
Longitudinal trajectories for voxel-wise metabolite ratios and conversion rates within the NAWM. The distribution of values within the ROI is summarized using the median. Values represent group-level averages of medians at each time point, computed across all patients contributing data for that treatment group and time point. Error bars denote the standard errors when more than one patient was available within the group at each time point. The number of patients contributing to each group-level estimate is indicated for BEV+, EVER+, and ALKYL+ at each time point (*N*).

Longitudinal comparison of metabolite ratios and conversion rates in the NAWM revealed distinct differences among patient groups, as shown in Fig. 2. Early changes over time in HP-^13^C metrics within the NAWM suggest increased relative lactate-to-pyruvate metabolism at the first follow-up for the *BEV+*, which showed an initial increase in lactate/pyruvate (average increase from baseline of 14.3%, at 1 month) and *k*_PL_ (average increase from baseline of 56.2% increase at 1 month) that then returned to normal by 2–5 months. In contrast, the *EVER+* group demonstrated a larger early decrease in lactate/pyruvate at 2 months (average decrease of 13.1%) and in *k*_PL_ at 2 months (average decrease of 22.2%), followed by partial recovery. These trends highlight distinct temporal patterns of HP-^13^C metabolism among the treatment groups, with *BEV+* showing early transient increases and *EVER+* presenting early decreases that partially normalize later.

### Voxel-wise Longitudinal Analysis of Representative Case Studies

**Figure 3:**
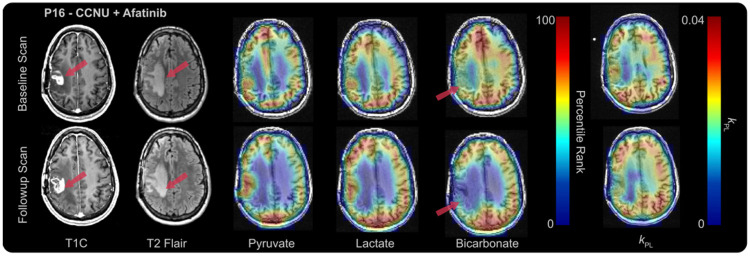
Representative slice for P16 at Pre-Tx (*top row*) and 2-month Post-Tx follow-up (*bottom row*). Post-contrast T1 and T2 FLAIR anatomical (*panels 1-2*), maps for Pyr%, Lac%, and Bic% (*panels 3-5*), and k_PL_ maps (panel 6) overlayed on the post-contrast T1 image. Arrows indicate T2 lesion.

**Figure 4:**
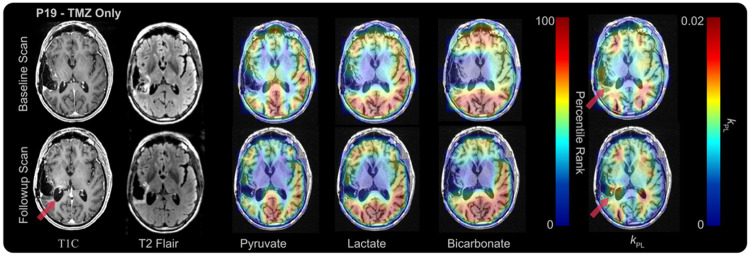
Representative slice for P19 at Pre-Tx (*top row*) and 1-month Post-Tx follow-up (*bottom row*). Post-contrast T1 and T2 FLAIR anatomical (panels 1-2), maps for Pyr%, Lac%, and Bic% (*panels 3-5*), and k_PL_ maps (*panel 6*) overlayed on the T1 image. Arrows indicate T2 lesion.

**Figure 5:**
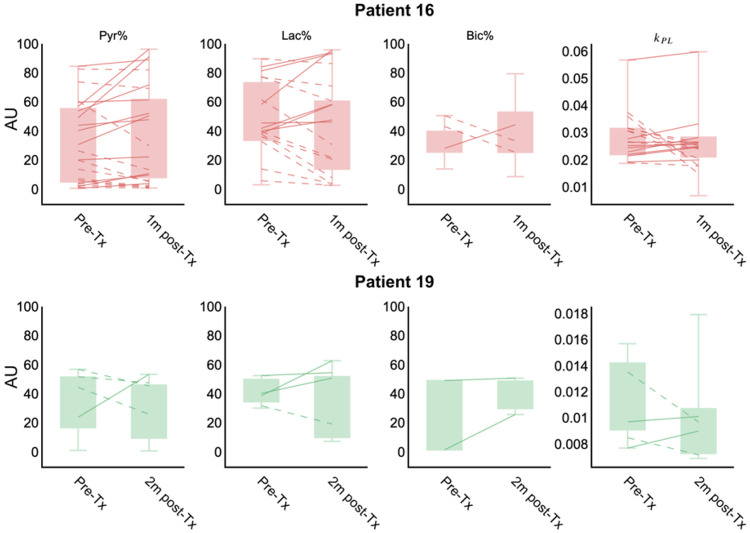
Voxel-wise comparisons between early and late progressors showing ranked metabolite percentile values (*panels 1–3*) and k_PL_ (*panel 4*) within T2L voxels for P16 (*top row*) and P19 (*bottom row*). Boxplots show the values within the ROI at each time point, while voxels connected by lines represent HP-^13^C voxels classified within the ROI at both time points for direct voxel-wise comparison. Solid lines indicate a voxel-wise increase, while dashed lines indicate a voxel-wise decrease.

Two patients, with data collected using the same templates^16^, were compared directly on a voxel-by-voxel basis: the first representing an early progressor (*P16*) and the second representing a late progressor (*P19*), as shown in Table 1. Representative slices for each patient at baseline and early post-treatment time points are shown in Figs. 3 and 4. Boxplots and voxel-wise comparisons within the T2L at follow-up time points, relative to the pre-treatment baseline, are shown in Fig. 5. In both patients, voxel-wise changes in Lac%, Pyr%, Bic%, and *k*_PL_ within the T2L, shown as voxels connected by lines, indicate varied treatment responses across tumors, while the changes in median values (boxplots) between the two time points remain small. Within voxels classified as within the T2L at both timepoints (Fig. 4, dashed and solid lines), P16 experienced an average of 9.4% decrease in Pyr%, a 7.7% decrease in Lac%, a 38.7% decrease in Bic% (n = 24 voxels for all metabolites), and a 10.1% decrease in k_PL_ (n = 17 voxels). Comparatively, P19, who experienced a longer PFS (9 months vs. 3 months), showed only a 2.61% decrease in Pyr%, a 14.72% increase in Lac%, a 12.94% increase in Bic%, and a 8.75% decrease in *k*_PL_ after 2 months of treatment (n = 4 voxels).

### Early Treatment Effects of Metabolism in the Lesion

**Figure 6:**
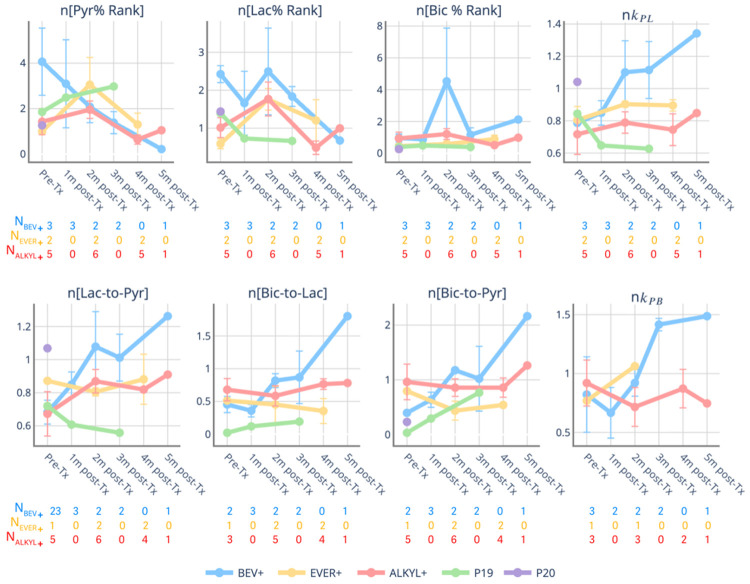
Longitudinal trajectories for voxel-wise ranked metabolite percentile statistics and ratios (*left*) and metabolite conversion rates (*right*) within the T2L. Distribution of normalized values within the ROI is summarized using the median values. Values represent group-level medians at each time point, computed across all patients contributing data to that treatment group and time point. Error bars denote the standard errors when more than one patient was available within the group at each time point. The number of patients contributing to each group-level estimate is indicated for *BEV*+, *EVER*+, and *ALKYL*+ at each time point. The number of patients contributing to each group-level estimate is indicated for BEV+, EVER+, and *ALKYL*+ at each time point (*N*).

**Figure 7:**
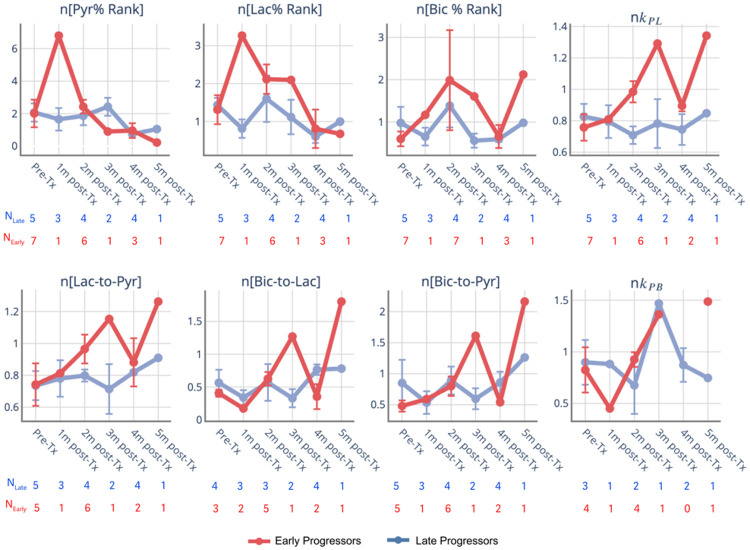
Longitudinal trajectories for voxel-wise ranked metabolite percentile and ratios (*left*) and metabolite conversion rates (*right*) within T2L. All the numbers are shown after patient-wise normalization by the NAWM median value (see Figure 2). The distribution of normalized values within the ROI is summarized by the median. Values represent group-level medians at each time point, computed across all patients contributing data to that treatment group and time point. Error bars denote the standard errors when more than one patient was available within the group at each time point. The number of patients contributing to each group-level estimate is indicated at each time point (*N*).

Figure 6 displays longitudinal changes in the normalized Pyr%, Lac%, Bic%, metabolite ratios, and conversion rates within the T2L voxels for each group, using patient-wise normalization based on the median values in the NAWM, given the treatment-induced changes illustrated in Fig. 2. Within the lesion, the initial change patterns are similar to those in the NAWM, with the *BEV+* increasing on Lac/Pyr (24.3% from baseline at 1 month) and the *EVER+* decreasing in Lac/Pyr (average of 7.6% from baseline at 2 months) after NAWM normalization. Figure 7 presents the same analyses after grouping patients by survival status. Among patients with early progression (PFS < 6 months), increases in Lac% (average of 148.8% increase from baseline), Lac/Pyr (average of 9.6% increase from baseline) and *k*_PL_ (average of 6.4% increase from baseline), and reductions in the Bic/Lac (average of 57.4% decrease from baseline) were observed as early as the 1-month follow-up. Although these values are averages across patients and driven by a single patient with T2L voxels at the 1-month timepoint, the trend toward elevated lactate production persists at 2 months following treatment. This pattern indicates a general treatment-related change in the distribution of pyruvate and lactate among early progressors. In contrast, patients without early progression demonstrate more modest longitudinal changes in Lac%, Lac/Pyr, and *k*_PL_ within T2L, indicating relatively stable metabolic profiles over time.

## Discussion

This study is the first to use serial HP [1-^13^C]pyruvate MRI to examine treatment response in patients with grade 4 glioma. The patients included in this study participated in both clinical trial and standard-of-care treatments, representing typical clinical practice. Treatment effects within the NAWM, depending on the type of therapy, have been observed as changes in metabolism. Additionally, early metabolite differences were identified in patients with early disease progression, offering valuable initial insights into the use of real-time metabolic imaging to evaluate treatment response in glioma.

Because of the heterogeneous treatment regimens used at the time of recurrence and prior work revealing metabolic changes on HP [1-^13^C]pyruvate MRI due to angiogenic^14^ and mTOR inhibitors^28^, subjects were grouped accordingly to facilitate analysis. Our analysis revealed treatment-dependent differences, most notably in the *BEV+* group. Patients treated with bevacizumab showed increases in Lac/Pyr and *k*_PL_ within the NAWM and T2L during early follow-up, as shown in Figs. 2 and 6. Changes in glucose and pyruvate metabolism were reported after anti-angiogenic therapy^29^, with greater uptake of labeled glucose and higher levels of labeled lactate in both tumors and the contralateral brain. Additionally, the antiangiogenic therapy induces hypoxia, leading to reduced perfusion^30^, as reflected by the decrease in %Pyr in T2L, which is consistent with prior research^14^. In the *EVER+* group, where mTOR inhibitors may affect brain metabolism by targeting core cellular functions, a shift from anabolic growth to catabolic maintenance occurs^31-33^. This is demonstrated by a reduction in lactate-to-pyruvate conversion in both NAWM and T2 lesions at the early time point. Although no statistically significant changes were observed in the normal brains of rats treated with Everolimus, the Lac/Pyr ratios in the tumors showed an early increase after normalization^28^, similar to the results shown in Fig. 6. While longitudinal changes were observed in the *ALKYL+*, these effects were less pronounced.

It is important to note that the relatively lower spatial resolution of HP-^13^C imaging may lead to partial volume effects, which can hinder the interpretation of results. Additionally, lesion size and location varied across patients, which complicated direct comparisons between subjects. By applying the prescription template in atlas space and then transforming it to the individual subject space^16^, we were able to achieve voxel-wise correspondence across time points for two patients. Such voxel-wise longitudinal comparisons in patients revealed significant spatial changes in metabolism at each time point between baseline and post-treatment follow-up scans. We particularly noted differences in parameter distributions between patients across T2L voxels. This underscores the diverse metabolic profiles both within tumors and among individuals and illustrates the difficulty of directly comparing HP-^13^C parameters across different subjects.

Patient-specific ranges of HP-^13^C metabolite parameters motivated the normalization of metabolite metrics relative to an internal NAWM reference before cross-patient longitudinal analysis of lesions. After this normalization, clearer patterns emerged when patients were grouped by PFS. Differences between early and late progressors became more evident at early post-treatment time points, as soon as 1–2 months, indicating that normalization improves sensitivity to progression-related metabolic changes, as shown in Fig. 7. These findings underscore the importance of patient-specific scaling in longitudinal and cross-patient analyses of HP-^13^C metabolic imaging data.

More importantly, similar to preclinical GBM models, where a decrease in lactate/pyruvate within 7–9 days after treatment with TMZ or mTOR inhibitors served as an early biomarker of treatment response^28, 34, 35^, we observed a pattern of an early increase in lactate/pyruvate and a decrease in the bicarbonate/lactate ratio after treatment in patients with shorter PFS. These findings provide initial insights into the early assessment of treatment response in high-grade glioma using HP [1-^13^C]pyruvate MRI. Monitoring metabolic changes in recurrent lesions from pretreatment to follow-up scans can indicate therapy effectiveness, guide treatment strategies, and influence clinical decisions.

Despite promising results from this initial longitudinal study using HP [1-^13^C]pyruvate MRI to evaluate treatment response, several limitations must be recognized. Although the cohort size is sufficient, variability in treatments restricted the depth of statistical analysis. Additionally, the low resolution of HP-^13^C imaging and the small size of post-surgical lesions led to fewer patients with follow-up data and usable voxels. Partial-volume effects, caused by the spatial constraints of HP-^13^C imaging, further hinder interpretation in small or heterogeneous lesions. Future research should aim to collect longitudinal data with higher temporal resolution to more effectively monitor response to treatment and include larger homogeneous cohorts to facilitate patient-specific intervention analysis.

## Figures and Tables

**Table 1:** Clinical summary of the patient population, including treatment regimens and scan intervals.

No	Age	Sex	Dx	Treatment	PFS6	OS (yr)	Baseline Acquired	Post- Tx F1 Scan
**1**	29	F	*rAS4*	**Bevacizumab** + [RT, TMZ]	1	0.7	No	2m, 3m, 5m
**2**	39	M	*rAS4*	**Bevacizumab**	0	5.0	No	1m
**3**	55	M	rGBM	**Bevacizumab** + [CCNU, Afatinib, Abemaciclib]	1	0.8	Yes	4m
**4**	63	M	rGBM	**Bevacizumab** + CCNU	0	0.9	Yes	1m, 3m
**5**	56	F	rGBM	**Bevacizumab** + [Carboplatin, Trametinib]	1	0.3	Yes	1m
**6**	55	M	rGBM	**Everolimus** + [CCNU, Trametinib, Abeciclib]	1	0.7	Yes	4m
**7**	62	M	rGBM	**Everolimus** + [TMZ, Olaparib]	1	0.8	No	2m
**8**	45	M	rGBM	**Everolimus** + [TMZ, Afatinib]	1	0.9	Yes	2m
**9**	68	M	rGBM	**Everolimus** + [CCNU, Dasatinib]	1	0.6	No	2m, 4m
**10**	33	M	*rAS4*	**TMZ** + [Olaparib, Abemaciclib]	0	1.4	No	2m, 3m
**11**	69	F	rGBM	**CCNU** + [Afatinib, Abemaciclib]	0	1.1	Yes	2m, 4m, 5m
**12**	57	M	rGBM	**TMZ** + [Afatinib, Olaparib]	0	3.7	No	2m, 4m
**13**	58	F	rGBM	**CCNU** + [Afatanib, Abecliclab]	0	0.4	Yes	2m
**14**	40	F	rGBM	**TMZ** + [Olaparib, Abemaciclib]	0	3.0	No	2m
**15**	57	M	rGBM	**TMZ** + [Propranolol,Trametinib,Abemaciclib]	1	1.1	Yes	4m
**16**	55	M	rGBM	**CCNU** + Afatinib	1	0.6	Yes	2m
**17**	55	F	rGBM	**TMZ** + [Afatinib, Olaparib]	0	1.6	Yes	4m
**18**	40	M	rGBM	**CCNU** + [Afatinib, Abemaciclib]	1	2.0	No	2m
**19**	59	F	rGBM	**TMZ**	0	1.1	Yes	1m, 3m
**20**	50	M	rGBM	**CCNU**	1	0.3	Yes	1m

rGBM: recurrent GBM; *rAS4: recurrent grade 4 astrocytoma*

## Data Availability

The processing scripts and example data will be made available by the authors. Further inquiries can be directed to the corresponding author.
